# Comparison of the surgical outcomes of free flap reconstruction for primary and recurrent head and neck cancers: a case-controlled propensity score-matched study of 1,791 free flap reconstructions

**DOI:** 10.1038/s41598-021-82034-5

**Published:** 2021-01-27

**Authors:** Kuan-Hua Chen, Spencer C. H. Kuo, Peng-Chen Chien, Hsiao-Yun Hsieh, Ching-Hua Hsieh

**Affiliations:** Department of Plastic Surgery, Kaohsiung Chang Gung Memorial Hospital, Chang Gung University and College of Medicine, No. 123, Ta-Pei Road, Niao-Song District, Kaohsiung City, 83301 Taiwan

**Keywords:** Diseases, Health care

## Abstract

This study was designed to compare the outcome and analyze the operation-related risk factors in free flap reconstruction for patients with primary and recurrent head and neck cancers. A 1:1 propensity score-matched analysis of the microsurgery registry database of the hospital. The primary outcome of the free flap reconstruction had a higher failure rate in the recurrent group than the primary group (5.1% vs. 3.1%, p = 0.037). Among the 345 pairs in the matched study population, there were no significant differences between the primary and recurrent groups regarding the rate of total flap loss (3.5% vs. 5.5%, p = 0.27) and secondary outcomes. This study revealed that free flap reconstruction had a higher failure rate in the recurrent group than the primary group, but such a difference may be attributed by the different patient characteristics.

## Introduction

Microvascular free flap reconstruction has been regarded as a standard procedure following head and neck cancer resection^1^. Its technique has progressed well over time and nowadays has achieved a success rate of 91–99%^2–4^. However, 50–60% of these patients develop a loco-regional recurrence within two years^5,6^ and eventually required a second or even more repetitive cancer resections and free flap reconstructions^7–9^. Because the previous surgery would result in scar tissue and possible anatomy changes, chemotherapy and concurrent radiotherapy may cause tissue change in the local region^10–12^, and the patient’s condition may be more vulnerable to future surgical procedures^13–15^. These patients with recurrent cancer are expected to have a lower success rate and higher risk of complication than those who undergo their first free flap reconstruction for primary head and neck cancer. However, such a hypothesis is yet validated in the literature. In addition, although many efforts have been devoted to clarifying the possible risk factors that were associated with the complications of free flap reconstruction^15,16^, exploration of the association of surgical complications with free flap reconstruction in primary or recurrent cancers was rather limited. Therefore, via a selected propensity-score matched population, this study aimed to investigate the outcome of free flap reconstruction following cancer resection for those patients with primary and recurrent head and neck cancer.

## Methods

This was a retrospective study and the work has been reported with the STROCSS criteria^17^. This study was approved with the reference number 201800440B0 by the institutional review board (IRB) of the Kaohsiung Chang Gung Memorial Hospital, a 2,686-bed medical center located in Kaohsiung city and serves as an important healthcare provider for patients in southern Taiwan^18–20^. According to IRB regulations, the requirement for informed consent was waived. All methods were performed in accordance with the relevant guidelines and regulations. We designed a retrospective study to review the microsurgery registry database of all 2,004 patients with head and neck cancer who underwent cancer resection and free flap reconstruction between March 2008 and February 2017. After excluding missing and incomplete data, 1,700 patients with a total of 1,791 free flap reconstructions (21 patients received double free flap reconstructions) were enrolled into the study. Detailed patient information was retrieved from medical records, including information regarding the following variables: age; sex; body mass index (BMI); status of alcohol drinking, betel nut chewing, and smoking; co-morbidities, such as diabetes mellitus (DM), hypertension (HTN), cerebrovascular accident (CVA), heart diseases (ICD-9 codes 402, 410–416, 420–429), renal diseases (ICD-9 codes 403–405, 580–589), and liver diseases (ICD-9 code 571); cancer stage groups (assigned as group 1 to 6) and locations (assigned as group 1 to 3) (Supplemental Table 1); preoperative chemotherapy and radiotherapy; flap types (anterolateral thigh (ALT), anteromedial thigh (AMT), freestyle, medial sural artery perforator (MSAP), fibula, and forearm), use of vein graft, contralateral microanastomosis, number of microanastomosis (1 artery and 1 vein, 1 artery and 2 veins, and 2 arteries and 2 veins); operator experience (years after getting a plastic surgeon board), and operation time (hours). Freestyle flaps are the flaps that were harvested in a free-style manner, once the perforator could be identified visually by doppler signals present in a specific region^21–23^. The primary outcome of this study was determined as the survival or failure of the flap, while the secondary outcomes were the associated complications, including wound infection, fistula, hematoma, and partial flap necrosis.

A comparison was made between the group of patients with primary cancer (n = 1,145) and the group of patients with recurrent cancer (n = 625). The collected data were compared using IBM SPSS Statistics for Windows version 23.0 (IBM Corp., Armonk, NY, USA). A two-sided Fisher’s exact test or Pearson Chi-square test was used to compare categorical data. The Mann–Whitney *U*-test was used to compare non-normally distributed data, which are presented as a median (interquartile range [IQR], Q1–Q3). Subsequently, to minimize the confounding effects due to a nonrandomized assignment in the evaluation of patient-related covariates, we created 1:1 propensity score-matched study population by the Greedy method using R software (version 3.5.0; package: Matchit, method: match it) calculated with a 0.2 caliper width to attenuate the influence of patient characteristics on the outcome assessment. The Greedy method is a matching algorithm widely applied by researchers to create a new sample of cases that share approximately similar likelihoods of being assigned to the treatment condition after obtaining estimated propensity scores. It selects a subject in primary group and then selects as a matched control subject, the subject in recurrent group whose propensity score is closest to that of the primary one. In the 1:1 ratio, if multiple subjects with recurrent tumor are equally close to the primary one, only one of these subjects with recurrent tumor is selected at random^24–26^. All results are presented as median with interquartile range (IQR, Q1-Q3) or number with percentage (n, %). A p-value of < 0.05 was considered statistically significant.

## Results

### Demographics of patient characteristics

The demographics of patient characteristics for patients with primary and recurrent head and neck cancer who received free flap reconstruction following cancer resection are summarized in Table 1. Compared to the patients with primary cancer, patients in the recurrent group were older, chewed betel nut, were diabetic and hypertensive, and underwent preoperative chemotherapy and radiotherapy. The cancer stages and locations were significantly different between the patients with primary and recurrent cancers. In terms of operation-related covariates, the flaps in the recurrent group had more contralateral microanastomosis (1.4% vs. 3.7%, p = 0.003) than those in the primary group. The types of flap and number of microanastomosis used in free flap reconstruction were different between the patients with primary and recurrent cancers. Among them, less ALT flaps (82.7% vs. 88.6%, p < 0.001) but more AMT flaps (5.1% vs. 1.8%, p < 0.001) and freestyle flaps (3.0% vs. 1.3%, p < 0.001) were used in patients with recurrent cancers than those with primary cancers. Reconstruction for recurrent cancer was performed by more experienced operators (median [IQR]; 3.6 years [1.2, 7.1] vs. 4.4 years [1.8, 10.4], p < 0.001). The operation time and the rate of vein graft use were not significantly different between these two groups (Table 1).

**Table 1:** Demographics of patients with primary and recurrent head and neck tumor who underwent free flap reconstruction following head and neck tumor resection.

	Primary n = 1,145	Recurrent n = 625	P-value
**Covariates, patient-related**			
Age (years, median [IQR])	53 [46,60]	57 [50,62]	< 0.001
Male gender (n, %)	1,093 (95.5)	595 (95.2)	0.814
BMI (median [IQR])	23.7 [21.0,26.6]	23.3 [20.8,26.1]	0.168
Alcohol (n, %)	940 (82.1)	530 (84.8)	0.164
Betel nut (n, %)	979 (85.5)	560 (89.6)	0.015
Smoking (n, %)	1,008 (88.0)	552 (88.3)	0.878
DM (n, %)	192 (16.8)	131 (21.0)	0.034
HTN (n, %)	294 (25.7)	201 (32.2)	0.004
CVA (n, %)	21 (1.8)	11 (1.8)	> 0.999
Heart disease (n, %)	60 (5.2)	36 (5.8)	0.661
Renal disease (n, %)	18 (1.6)	9 (1.4)	> 0.999
Liver disease (n, %)	61 (5.3)	32 (5.1)	0.911
**Tumor stage groups (n, %)**			< 0.001
1	126 (11.0)	108 (17.3)	
2	190 (16.6)	132 (21.1)	
3	69 (6.0)	19 (3.0)	
4	259 (22.6)	217 (34.7)	
5	495 (43.2)	145 (23.2)	
6	6 (0.5)	4 (0.6)	
**Tumor locations (n, %)**			< 0.001
1	613 (53.5)	389 (62.2)	
2	371 (32.4)	156 (25.0)	
3	161 (14.1)	80 (12.8)	
Radiotherapy (n, %)	162 (14.1)	370 (59.2)	< 0.001
Chemotherapy (n, %)	202 (17.6)	317 (50.7)	< 0.001
**Covariates, operation-related**			
Use of vein graft (n, %)	11 (1.0)	9 (1.4)	0.357
Contralateral microanastomosis (n, %)	16 (1.4)	23 (3.7)	0.003
**Flap types (n, %)**			< 0.001
ALT	1,015 (88.6)	517 (82.7)	
AMT	21 (1.8)	32 (5.1)	
Freestyle	15 (1.3)	19 (3.0)	
MSAP	7 (0.6)	4 (0.6)	
Fibula	70 (6.1)	39 (6.2)	
Forearm	17 (1.5)	14 (2.2)	
**Number of microanastomosis (n, %)**			< 0.001
1 artery, 1 vein	853 (74.5)	527 (84.3)	
1 artery, 2 veins	282 (24.6)	98 (15.7)	
2 arteries, 2 veins	10 (0.9)	0 (0.0)	
Operator experience (years, median [IQR])	3.6 [1.2,7.1]	4.4 [1.8,10.4]	< 0.001
Operation time (hours, median [IQR])	7.1 [5.8,8.6]	7.2 [5.9,8.6]	0.314

ALT = anterior lateral thigh; AMT = anterior medial thigh; BMI = body mass index; DM = diabetes mellitus; HTN = hypertension; CVA = cerebrovascular accident; IQR = interquartile range; MSAP = medial sural artery perforator.

### Surgical outcomes of the unmatched and matched groups of patients

As shown in Table 2, the primary outcome of free flap reconstruction had a higher failure rate in the recurrent group than the primary group (5.1% vs. 3.1%, p = 0.037). Among 1145 patients with primary cancer, 35 of which had a total flap loss in the free flap reconstruction, whereas 32 out of 625 recurrent patients underwent a failed reconstruction surgery. However, there were no significant differences between the two groups of patients regarding the secondary outcomes of wound infection rate, fistula, hematoma, and partial necrosis of the flap. Further, a 1:1 propensity score-matched study population was created by including 345 well-balanced pairs by adjusting all patient-related covariates, including age, betel nut use, DM, hypertension, tumor stage groups, tumor locations, and radiotherapy.

**Table 2:** Comparison of outcomes between the primary and recurrent groups after propensity score matching of patient-related factors.

	Primary n = 1,145	Recurrent n = 625	P-value
**Outcomes, before matching**			
Total flap loss (n, %)	35 (3.1)	32 (5.1)	0.037
Wound infection (n, %)	272 (23.8)	133 (21.3)	0.261
Fistula (n, %)	106 (9.3)	54 (8.6)	0.729
Hematoma (n, %)	69 (6.0)	37 (5.9)	> 0.999
Partial necrosis (n, %)	149 (13.0)	92 (14.7)	0.346

	Primary n = 345	Recurrent n = 345	P-value
**Outcomes, after matching patient-related factors**			
Total flap loss (n, %)	12 (3.5)	19 (5.5)	0.27
Wound infection (n, %)	84 (24.3)	84 (24.3)	> 0.999
Fistula (n, %)	32 (9.3)	33 (9.6)	> 0.999
Hematoma (n, %)	16 (4.6)	22 (6.4)	0.404
Partial necrosis (n, %)	51 (14.8)	51 (14.8)	> 0.999

These matched groups of patients did not present significantly different patient-related covariates (Supplemental Table 2). Among these 345 pairs of matched subjects, there were no significant differences between the primary and recurrent groups regarding the primary outcome, the rate of total flap loss (3.5% vs. 5.5%, p = 0.27), and secondary outcomes (wound infection, fistula, hematoma, and partial necrosis).

### Operation-related covariates of the matched groups of patients

Among these 345 pairs, there were more contralateral microanastomosis in the recurrent group (1.4% vs. 4.6%, p = 0.024). The types of flaps were different between patients with primary and recurrent cancers. Less ALT flaps (83.2% vs. 91.3%, p < 0.001) but more AMT flaps (5.5% vs. 1.4%, p < 0.001) and MSAP flaps (6.7% vs. 2.3%, p < 0.001) were used in patients with recurrent cancers than those with primary cancers. The rate of vein graft use, the number of microanastomosis, the operator’s experience, and operation time were not significantly different between the two groups (Table 3).

**Table 3:** Comparison between the primary and recurrent groups after propensity score matching of patient-related covariates.

	Primary n = 345	Recurrent n = 345	P-value
Use of vein graft (n, %)	1 (0.3)	2 (0.6)	> 0.999
Contralateral microanastomosis (n, %)	5 (1.4)	16 (4.6)	0.024
**Flap types (n, %)**			0.004
ALT	315 (91.3)	287 (83.2)	
AMT	5 (1.4)	19 (5.5)	
Freestyle	8 (2.3)	9 (2.6)	
MSAP	8 (2.3)	23 (6.7)	
Fibula	6 (1.7)	5 (1.4)	
Forearm	3 (0.9)	2 (0.6)	
**Number of microanastomosis (n, %)**			0.169
1 artery, 1 vein	274 (79.4)	284 (82.3)	
1 artery, 2 veins	68 (19.7)	61 (17.7)	
2 arteries, 2 veins	3 (0.9)	0 (0.0)	
Operator experience (years, median [IQR])	4.1[1.4,7.9]	4.38[1.6,9.8]	0.233
Operation time (hours, median [IQR])	7.2[5.8,8.7]	7.1[5.8,8.6]	0.92

ALT = anterior lateral thigh; AMT = anterior medial thigh; IQR = interquartile range; MSAP = medial sural artery perforator.

## Discussion

In this study, there was significantly different use of flap types for reconstruction in patients with primary and recurrent cancers either before or after propensity score matching. Notably, less ALT flaps were used for patients with recurrent cancers than patients with primary cancers. This may be attributed to the fact that the ALT flap has generally been the first choice and is used for reconstruction in prior surgery^27,28^, leaving the surgeons to no longer choose an anterolateral thigh flap, even from the contralateral thigh, for further free flap reconstruction following repeated cancer resection. This is also reflected by the more frequently used AMT flap and that the freestyle flap was used in patients with recurrent cancers compared to those with primary cancers.

The use of vein grafts has been associated with significantly higher flap losses than those with direct microanastomosis^29^. Although the most common scenario in which vein grafting is required is the lack of nearby or even ipsilateral healthy recipient vessels, and such conditions were generally encountered in those who had a prior failed reconstruction, a repeated cancer resection, or those who already received radical neck dissection or radiotherapy^16^. However, in this study, there was no significant difference of vein graft use between those who had primary or recurrent cancers either before or after propensity score matching. In contrast, there was a higher rate of contralateral microanastomosis of the patients with recurrent cancers than those with primary cancers during free flap reconstruction either before or after propensity score matching. This might be contributed by the fact that the vessels nearby have already been used in a previous reconstruction surgery for the patient’s primary cancer^30,31^. Since vascular microanastomosis is the critical step for a successful free flap transfer^32,33^, the surgeons might have foreseen the disadvantages of using the vessels in the same side in a prior surgery or radiotherapy and also choose not to use a vein graft but rather proceed with contralateral microanastomosis instead. Accordingly, the reconstructive option seemed to be different in handling the recurrent cancers and the primary cancers.

In this study, reconstructions for the recurrent cancers were performed by surgeons with more experience than those in the primary cancers. This study also revealed that the free flap reconstruction had a higher failure rate in the recurrent group than the primary group, but this difference was not found in the matched groups of patients who had similar patient-related covariates. This result implied that the higher failure rate of the free flap reconstruction in patients with recurrent cancers than primary cancers may be mostly attributed by the different patient characteristics. The above observation may also sketch a situation that, in the reconstruction of the recurrent cancers, the experienced surgeons may choose an alternative flap other than the ALT flap for reconstruction, prefer contralateral microanastomosis, but somehow intentionally not to use the vein grafts to prevent anticipated challenges. However, such a hypothesis requires more studies for validation, seeing there are still debates regarding the successful rates of different flap types^3,34^ and the altitude and circumstance in doing the reconstruction for patients with primary or recurrent cancers were unknown.

In this study, the use of propensity score-matching analysis presented a specific strength to markedly reduce bias on covariate analysis. However, this study also has a number of limitations. First, we must consider the inherent bias of the retrospective studies. For example, the indication of flap use and the reconstruction option may vary among different surgeons^35,36^. Second, it is not likely to guarantee an even distribution of unmeasured confounders between the primary and recurrent groups. For example, patients with recurrent cancers may have had a worse nutrition status or immunocompromised status that caused their cancer to recur^8,37^, their vessels conditions and hemostasis may also be worse than the patients with primary cancers^38^. In addition, the selection of matched study population in this study only represented 55.2% (345/625) of the patients with recurrent cancers, indicating a remarked difference of patient characteristics between the patients with primary and recurrent cancers, which may lead to some selection bias in the data analysis. Last, the study population was limited to a single urban medical center in southern Taiwan, which may not be representative of other populations. A more comprehensive, prospective, and protocol-based study is needed to address these issues.

## Conclusion

This study revealed that the free flap reconstruction had higher failure rate in the recurrent group than the primary group, but such a difference may be mostly attributed by the different patient characteristics. In addition, the reconstructive option determined by the surgeons seemed to vary in handling recurrent and primary cancers.

## Supplementary Information

Supplementary Table 1.

Supplementary Table 2.
