# Supplementary material for: Comparison of the surgical outcomes of free flap reconstruction for primary and recurrent head and neck cancers: a case-controlled propensity score-matched study of 1,791 free flap reconstructions
Source: Sci Rep. 2021 Jan 27;11:2350. doi: 10.1038/s41598-021-82034-5 (PMC7840944; doi:10.1038/s41598-021-82034-5)
Supplement: Supplementary file 1 — Supplementary Table 1. [file 41598_2021_82034_MOESM1_ESM.docx]

**Comparison of the surgical outcomes of free flap reconstruction for primary and recurrent head and neck cancers:** **a case-controlled propensity score-matched study of 1,791 free flap reconstructions**

Kuan-Hua Chen, Spencer CH Kuo, Peng-Chen Chien, Hsiao-Yun Hsieh, Ching-Hua Hsieh*

Department of Plastic Surgery, Kaohsiung Chang Gung Memorial Hospital, Chang Gung University and College of Medicine, Kaohsiung 83301, Taiwan

Kuan-Hua Chen; wilbertrock@gmail.com

Spencer C.H. Kuo; spenc19900603@gmail.com

Peng-Chen Chien; [venu_chien@hotmail.com](mailto:venu_chien@hotmail.com)

Hsiao-Yun Hsieh; sylvia19870714@hotmail.com

Ching-Hua Hsieh; m93chinghua@gmail.com

Corresponding author: Ching-Hua Hsieh, M.D., PhD, FACS

Department of Plastic Surgery, Kaohsiung Chang Gung Memorial Hospital and Chang Gung University College of Medicine, Taiwan

No.123, Ta-Pei Road, Niao-Song District, Kaohsiung City 833, Taiwan

**Supplemental Table 1.** Definition of tumor stage groups and tumor locations

| Tumor stage groups | | Tumor locations | |
| --- | --- | --- | --- |
| 1 | T = 1, N = 0, M = 0 | 1 | Buccal/palate/lip/gum |
| 2 | T = 2, N = 0, M = 0 | 2 | Mouth floor/tongue/trigon/tongue base/tonsil |
| 3 | T = 3, N = 0, M = 0 | 3 | Oropharygeal/hypopharyngeal |
| 4 | T = 4, N = 0, M = 0 |  |  |
| 5 | Any T, N ≥ 1, M = 0 |  |  |
| 6 | Any T, Any N, M ≥ 1 |  |  |
