# Supplementary material for: Comparison of the surgical outcomes of free flap reconstruction for primary and recurrent head and neck cancers: a case-controlled propensity score-matched study of 1,791 free flap reconstructions
Source: Sci Rep. 2021 Jan 27;11:2350. doi: 10.1038/s41598-021-82034-5 (PMC7840944; doi:10.1038/s41598-021-82034-5)
Supplement: Supplementary file 2 — Supplementary Table 2. [file 41598_2021_82034_MOESM2_ESM.docx]

**Comparison of the surgical outcomes of free flap reconstruction for primary and recurrent head and neck cancers:** **a case-controlled propensity score-matched study of 1,791 free flap reconstructions**

Kuan-Hua Chen, Spencer CH Kuo, Peng-Chen Chien, Hsiao-Yun Hsieh, Ching-Hua Hsieh*

Department of Plastic Surgery, Kaohsiung Chang Gung Memorial Hospital, Chang Gung University and College of Medicine, Kaohsiung 83301, Taiwan

Kuan-Hua Chen; wilbertrock@gmail.com

Spencer C.H. Kuo; spenc19900603@gmail.com

Peng-Chen Chien; [venu_chien@hotmail.com](mailto:venu_chien@hotmail.com)

Hsiao-Yun Hsieh; sylvia19870714@hotmail.com

Ching-Hua Hsieh; m93chinghua@gmail.com

Corresponding author: Ching-Hua Hsieh, M.D., PhD, FACS

Department of Plastic Surgery, Kaohsiung Chang Gung Memorial Hospital and Chang Gung University College of Medicine, Taiwan

No.123, Ta-Pei Road, Niao-Song District, Kaohsiung City 833, Taiwan

**Supplemental Table 2.** The demographic profile in the selected studied population with matched patient-related factors

|  | **Primary**  **n=345** | **Recurrent**  **n=345** | **P-value** |
| --- | --- | --- | --- |
| Age (years, median [IQR]) | 55 [49, 61] | 56 [49, 61] | 0.515 |
| Male gender (n, %) | 330 (95.7) | 325 (94.2) | 0.488 |
| BMI (median [IQR]) | 23.53 [20.66, 26.24] | 23.57 [20.87, 26.53] | 0.542 |
| Alcohol (n, %) | 286 (82.9) | 286 (82.9) | >0.999 |
| Betel nut (n, %) | 306 (88.7) | 303 (87.8) | 0.813 |
| Smoking (n, %) | 306 (88.7) | 302 (87.5) | 0.724 |
| DM (n, %) | 62 (18.0) | 71 (20.6) | 0.44 |
| HTN (n, %) | 100 (29.0) | 107 (31.0) | 0.618 |
| CVA (n, %) | 8 (2.3) | 6 (1.7) | 0.788 |
| Heart disease (n, %) | 16 (4.6) | 18 (5.2) | 0.861 |
| Renal disease (n, %) | 4 (1.2) | 5 (1.4) | >0.999 |
| Liver disease (n, %) | 15 (4.3) | 20 (5.8) | 0.488 |
| Tumor stage groups (n, %) |  |  | 0.15 |
| 1 | 57 (16.5) | 58 (16.8) |  |
| 2 | 61 (17.7) | 69 (20.0) |  |
| 3 | 26 (7.5) | 13 (3.8) |  |
| 4 | 79 (22.9) | 97 (28.1) |  |
| 5 | 119 (34.5) | 106 (30.7) |  |
| 6 | 3 (0.9) | 2 (0.6) |  |
| Tumor locations (n, %) |  |  | 0.996 |
| 1 | 200 (58.0) | 201 (58.3) |  |
| 2 | 93 (27.0) | 92 (26.7) |  |
| 3 | 52 (15.1) | 52 (15.1) |  |
| Radiotherapy (n, %) | 134 (38.8) | 133 (38.6) | >0.999 |
| Chemotherapy (n, %) | 139 (40.3) | 127 (36.8) | 0.39 |

BMI = body mass index; DM = diabetes mellitus; HTN = hypertension; CVA = cerebrovascular accident; IQR = interquartile range.
